# Supplementary material for: Effect of therapeutic hypothermia on renal and myocardial function in asphyxiated (near) term neonates: A systematic review and meta-analysis
Source: PLoS One. 2021 Feb 25;16(2):e0247403. doi: 10.1371/journal.pone.0247403 (PMC7906340; doi:10.1371/journal.pone.0247403)
Supplement: S1 Text — (DOCX) [file pone.0247403.s002.docx]

# **S1 Text. Full search strategy.**

The exact search for kidney failure used in PubMed was: ("hypothermia, induced"(mesh) OR hypothermia OR cooling) AND ("infant, newborn"(mesh) OR infant OR newborn OR neonate) AND (“kidney failure, chronic”(mesh) OR “renal insufficiency, chronic”(mesh) OR “acute kidney injury”(mesh) OR “acute kidney” OR “kidney” OR "dialysis" OR “renal”) AND (“asphyxia”(mesh) OR “hypoxia”(mesh) OR “hypoxia-ischemia, brain”(mesh) OR “hypoxia, brain”(mesh) OR “ischemia”(mesh) OR “hypoxic ischemic encephalopathy” OR “asphyxia” OR “hypoxia” OR “ischemia” OR “aortic arch” OR “shock” OR “ near-drowning” OR drown*) AND (“long-term” OR “long term” OR “chronic” OR “follow up” OR “follow-up” OR “development” OR “outcome” OR “prognosis”)

No limitation in search field was used in this search.

The exact search for cardiac injury used in Pubmed was: ("hypothermia, induced"(mesh) OR hypothermia OR cooling) AND ("infant, newborn"(mesh) OR infant OR newborn OR neonate) AND ("asphyxia"(mesh) OR asphyxia OR "hypoxia"(mesh) OR "hypoxia-Ischemia, brain"(mesh) OR "hypoxia, brain"(mesh) OR “hypoxic ischemic encephalopathy” OR hypoxia OR “ischemia”(mesh) OR “ischemia” OR “aortic arch” OR “shock” OR “ near-drowning” OR drown*) AND (“heart failure”(mesh) OR “heart”(mesh) OR “heart failure” OR myocard* OR cardiac OR heart OR troponin OR “CPK-MB” OR “CK-MB”) AND (“long-term” OR “long term” OR “chronic” OR “follow up” OR “follow-up” OR “development” OR “outcome” OR “prognosis”)

No limitation in search field was used in this search.
