# Supplementary material for: Effect of therapeutic hypothermia on renal and myocardial function in asphyxiated (near) term neonates: A systematic review and meta-analysis
Source: PLoS One. 2021 Feb 25;16(2):e0247403. doi: 10.1371/journal.pone.0247403 (PMC7906340; doi:10.1371/journal.pone.0247403)
Supplement: Attachment — Submitted filename: Response to Reviewers.docx [file pone.0247403.s004.docx]

# Response to reviewers

Karel Allegaert

PLOS Headquarters: 1265 Battery Street, San Francisco, CA 94111, United States

Utrecht, January 23, 2021

Re: PONE-D-20-36313

Dear colleague,

We are pleased to resubmit our revised manuscript < Effect of therapeutic hypothermia on renal and myocardial function in asphyxiated (near) term neonates: a systematic review and meta-analysis> to PLoS One.

We thank the editor and the reviewers for their valuable comments, and we have revised the manuscript to address the questions raised.

Please find below our responses to the reviewers in detail.

All authors have read and approved the revised version of the mansucript. The manuscript has not been submitted elsewhere.

We hope that our manuscript will be considered for publication in PLoS One.

Sincerely,

On behalf of all authors,

Karen de Bijl-Marcus

## Journal requirements

**When submitting your revision, we need you to address these additional requirements.**

**1. Please ensure that your manuscript meets PLOS ONE's style requirements, including those for file naming. The PLOS ONE style templates can be found at**

[**https://journals.plos.org/plosone/s/file?id=wjVg/PLOSOne_formatting_sample_main_body.pdf**](https://journals.plos.org/plosone/s/file?id=wjVg/PLOSOne_formatting_sample_main_body.pdf) **and**

[**https://journals.plos.org/plosone/s/file?id=ba62/PLOSOne_formatting_sample_title_authors_affiliations.pdf**](https://journals.plos.org/plosone/s/file?id=ba62/PLOSOne_formatting_sample_title_authors_affiliations.pdf)

We have checked the templates and adapted the headings to meet the style requirements. We also changed the ‘:’ in the table titles to a ‘.’. The references are cited in order of first mention.

The file naming is according to the requirements.

In the title page, we now listed the corresponding author’s initials in parentheses after the email address and put the ‘¶’ in superscript.

We used PACE to check our figures and converted them to accepted formats.

We believe the manuscript now meets all the style requirements. Please let us now if we did still overlook something.

**2. Please provide an analysis of publication bias also known as sensitivity analysis on the studies included in the systematic review.**

Reviewer 1 pointed out that publication bias tests and plots are only relevant if you have >10 studies. We added a sentence to the method section on the fact that Egger’s test to investigate publication bias will only be performed if >10 studies are included (line 149) and explained that it was not appropriate to assess publication bias in our result section (line 249). We added this as a limitation to our discussion section (line 393).

## Reviewer #1 comments:

**I will focus on methods and reporting.
Quallity assessment is appropriate.**

Thank you for your very helpful comments on especially the methods and reporting. **Major
1) Avoid fixed effect models since they under-perform in the presence of ANY heterogeneity. Random-effects (RE) models are more conservative and provide better estimates with wider confidence intervals:** [**http://www.ncbi.nlm.nih.gov/pubmed/11252006**](http://www.ncbi.nlm.nih.gov/pubmed/11252006) **and** [**http://www.ncbi.nlm.nih.gov/pubmed/21148194**](http://www.ncbi.nlm.nih.gov/pubmed/21148194)**. What does the arbitrary 50% cut-off point add? Why is 49% fine and 51% an issue? My point is that a RE model will work better, in the presence of 5% heterogeneity, compared to a FE model!**

We agree. Instead of choosing between a fixed effect and a random effects model on the basis of the test for heterogeneity, we now chose to use the DerSimonian and Laird random effects model as the presence of heterogeneity between the studies was very likely.

**2) Report the confidence intervals for I^2 (calculated using heterogi or metaan in Stata) as argued in** [**http://www.ncbi.nlm.nih.gov/pubmed/17974687**](http://www.ncbi.nlm.nih.gov/pubmed/17974687)**. A simple formula exists in the seminal 2002 Higgins paper that proposed I^2.**

We have calculated the confidence intervals for I^2^ and reported these in the manuscript (line 247). In our discussion, we added a phrase on the interpretation of the presence of heterogeneity in our manuscript (line 401).

**Minor
1) Abstract: meta analysis methods should be included in the abstract i..e random or fixed effects modelling, how was heterogeneity quantified, publication bias assessed etc.**

We added the meta-analysis methods to the abstract (line 38).

**2) Do not use the term WMD, it is confusing. Say MD instead. Please see** [**http://handbook.cochrane.org/chapter_9/9_2_3_1_the_mean_difference_or_difference_in_means.htm**](http://handbook.cochrane.org/chapter_9/9_2_3_1_the_mean_difference_or_difference_in_means.htm)

We rephrased the term “weighted mean difference (WMD)” to “mean difference (MD)” (line 143).

**3) I^ is not a test but a statistic.**

We rephrased “the I^2^ test” to “the statistic i^2^” (line 148).

**4) Publication bias tests and plots only relevant if you have >10 studies otherwise underpowered to detect much and tend to lead to conclusions that are not justified** [**http://www.ncbi.nlm.nih.gov/pubmed/11106885**](http://www.ncbi.nlm.nih.gov/pubmed/11106885)**. If you don’t have enough studies to assess you should discuss this as a major limitation. Even with 10 or 20 studies it is very difficult to visually assess. If you have 20 or more studies it is a considerable strength.**

We agree and added a phrase on this limitation to our discussion (line 393).

**5) Year may be worth considering in bias assessment, especially if you don't have enough studies for a formal test:** [**http://www.ncbi.nlm.nih.gov/pubmed/25988604**](http://www.ncbi.nlm.nih.gov/pubmed/25988604)**. With newer studies we would be more confident.**

We added a phrase on the year of the studies and the relevance of more recent studies to our discussion (line 396).

**6) Note that MH is traditionally a fixed effect approach and the random effects version in RevMan is an IV-MH hybrid method. Some clarification on the MH weighting is needed (it is inverse variance as well, like your analysis of the continiuous outcomes).**

We agree that the DerSimonian and Laird inverse variance method is a better approach than the MH method. We have now performed the meta-analysis using the DerSimonian and Laird method and changed this in our method section (line 145). The meta-analysis presented in figure 3 is now the following:

**7) How was the random-effect model implemented, i.e. how was heterogeneity estimated? There are numerous ways to do so. Did they use the standard DerSimonian-Laird method? If so, please state so. Also there are better performing methods, for example please see** [**https://www.ncbi.nlm.nih.gov/pubmed/28815652**](https://www.ncbi.nlm.nih.gov/pubmed/28815652) **(or** [**http://www.ncbi.nlm.nih.gov/pubmed/23922860**](http://www.ncbi.nlm.nih.gov/pubmed/23922860)**) and the metaan command in Stata where these are implemented (**[**https://www.stata-journal.com/article.html?article=st0201**](https://www.stata-journal.com/article.html?article=st0201)**).**

**Cochran Q (i.e. chi-square) is notoriously underpowered to detect heterogeneity, especially for small meta-analyses** [**http://www.ncbi.nlm.nih.gov/pubmed/9595615**](http://www.ncbi.nlm.nih.gov/pubmed/9595615)**. I would not use.**

**Regarding heterogeneity estimates, all these estimates are very likely off, especially for small meta-analyses, and you should be wary about homogeneity assumptions** [**http://www.ncbi.nlm.nih.gov/pubmed/23922860**](http://www.ncbi.nlm.nih.gov/pubmed/23922860)**.**

We now clarified that we used the DerSimonian-Laird method and the I^2^ statistic to estimate heterogeneity (line 145-149). We also added the 95% CI of I^2^ (line 148). In our discussion, we added a phrase on the interpretation of the presence of heterogeneity in our manuscript (line 404).

## Reviewer #2 comments:

**Comments on the manuscript # PONE-D-20-36313 entitled «The long-term effects of therapeutic hypothermia on renal and myocardial function in asphyxiated full-term neonates: a systematic review and meta-analysis»

This is a really interesting systematic review and meta-analysis on the possible protective effect on renal and myocardial function of infants undergoing TH. According to the results, TH in asphyxiated neonates reduces the incidence of AKI, thus potentially being renoprotective and, also, might have a cardioprotective effect on the short-term.
In general, this is a well written paper and, I only have minor comments. The language is satisfactory.**

Thank you for your very helpful comments.

**Title
I think that the title should change to «Effect of therapeutic hypothermia on renal and myocardial function in asphyxiated neonates: a systematic review and meta-analysis».
The reasons why I am suggesting this change is due to the fact that a) the impact of TH on myocardial dysfunction was not made possible to be evaluated in the long-term, b) mostly short-term outcomes were studied, and c) TH includes both near-term and term infants (this should be taken into consideration in the whole manuscript).**

We agree with the arguments for adapting the title and changed it to “Effect of therapeutic hypothermia on renal and myocardial function in asphyxiated (near) term neonates: a systematic review and meta-analysis.”

**Abstract
1. Objective: it should be: to evaluate the short- and long-term effects of TH on renal and myocardial function in asphyxiated neonates.**

We agree and rephrased “full-term” to “(near) term”, since the included studies included neonates born at >35 weeks (line 32). We also made this change in other parts of the manuscript (lines 83, 91).

**Introduction
1. L76. Please add the “possible” short- and long-term effects.**

We added “possible” to this line (line 82).

**Methods – Search strategy
1. L94: ….to possibly identify a valid predictor…! I am not sure what the meaning of this statement is! Do authors try to identify any predictor of renal or cardiac dysfunction? Please clarify!**

We rephrased “to possibly identify a valid predictor on the long-term effect” to “to possibly identify studies on outcomes that indicated predictors of long-term outcome” (line 99). We hope this is more clear.
 **2. L105: A relevant comment regarding criterion # 5! Please, rephase or clarify!**

We rephrased this sentence to “long-term outcome or a valid marker of long-term outcome must be reported.” (line 114). **3. L113: Please change reviewer to investigator of researcher.**

We changed “reviewer” to “researcher” (line 122).

**Results
1. L146-147: Please, delete the phrase “Two different ….” As this is already mentioned in the method section.**

We deleted this sentence. **2. L172: it is written that “There were no significant differences in baseline characteristics between the TH group compared to the control group in 7 studies”. How is it concluded? Please, delete or rephrase the statement.**

The absence or presence of significant differences in baseline characteristics were presented in the articles of the included studies, a P-value less than 0.05 was considered significant.

We rephrased this sentence (line 178).

**3. L267: As before, delete the sentence regarding the 2 searchers…**

We deleted this sentence. **4. L320: Apparently the aim of the study was to assess both short- and long-term effects of TH on the kidneys.**

We changed “… long-term effect…” to “short-term and long-term effects” (line 315). We also changed “.. a search..” to “..two searches..” to make this more clear (line 314). **Discussion
It does provide a satisfactory explanation of the results, although it could be slightly shortened.**

Thank you, we have shortened and deleted some parts of the discussion.

**1. L 322: it is said that “…a review and meta-analysis of studies on the incidence of AKI after treatment with TH was performed”. Generally speaking, I believe that the terms “short- and long-term” should be more clearly specified in the present document (in possibly in the method?). Therefore, at this point it would be better to state “the incidence of AKI during the first week of life” instead to after treatment which is rather indefinite and general.**

We added a specification on the terms ‘short-term’ and ‘long-term’ to the method section (line 100).

Because some of the included studies did not specify on the time of measurement of the renal parameters, we can not be sure that the diagnosis of AKI was made in the first week of life for all studies. Therefore, we added “during the hospital course in the first period of life” to the sentence instead of “during the first week of life” (line 317).

Furthermore, the purpose of this sentence was to state that we compared the incidence of AKI after treatment with TH to the incidence in the control groups, and not to indicate a specific time period. Therefore, we did not remove “after treatment with TH in comparison to control groups without TH.” We do think that the sentence now does more clearly specify that the incidence of AKI was measured on the short-term, relatively close after birth. **2. L352: what does RAAS stand for?**

We added the full term “renin-angiotensin-aldosteron system” and put the abbreviation in brackets (line 347).

### Reviewer #3 comments:

**Thank you for the invitation to review the Manuscript PONE-D-20-36313
"The long-term effects of therapeutic hypothermia on renal and myocardial function in asphyxiated full-term neonates: a systematic review and meta-analysis"for PLOS ONE.

In this systematic review, the authors reported the short- and long-term effects of therapeutic hypothermia (TH) on renal and myocardial function in asphyxiated full-term neonates. Therefore, the data was obtained from an electronic search strategy incorporating MeSH terms and keywords in October 2019 and updated in June 2020 using PubMed and Cochrane databases. Inclusion criteria consisted of an RCT or observational cohort design.
They mainly identified nine studies which described the effect of TH on the incidence of acute kidney injury (AKI) after perinatal asphyxia. Meta-analysis showed a significant difference between the incidence of AKI in neonates treated with TH compared to the control group.
The authors concluded that TH in asphyxiated neonates reduces the incidence of AKI. No studies were found which investigated the effects of TH on long-term renal function or myocardial function. Only few studies suggest short-term beneficial effects on myocardial function.

General comments

The study is generally well written and methodologically sound.
The abstract has a clear objective. The introduction is clear and the aim is well defined. The main strength of the study is that the authors report extensively on the search strategy, study selection and quality assessment with the main result of protective effects on renal function in 9 studies in TH treated asphyxiated neonates which were included in the meta-analysis.
The weakness of the systemic review is that the authors did not find any studies which describe the long-term effects on renal or myocardial function.**

Thank you for your very helpful comments. We agree that our review is limited because of the absence of studies on the long-term effects, we have tried to emphasize the need for further research on this topic.

**I'm not convinced that the description of the values in the text adds to what's already presented in the Figures, in fact there's unnecessary repetition between text and figures.**

We shortened the text on the systematic search (lines 167 and 269) as this is already presented in figure 1 and we shortened the legend of figure 3 (line 256).

**Furthermore, I am wondering that the authors did not consider blood pressure and the use of catecholamines or volume therapy when assessing kidney function**

We chose to use serum creatinine and urine output to assess kidney function as these parameters are used in the KDIGO guideline definition for AKI. The KDIGO guideline provides the following argument: “It is widely accepted that GFR is the most useful overall index of kidney function in health and disease, and changes in SCr and urine output are surrogates for changes in GFR. In clinical practice, an abrupt decline in GFR is assessed from an increase in SCr or oliguria.” Furthermore, several studies have shown that AKI is a risk factor for CKD and therefore serves as a predictor of the long-term renal effect.

**I have several specific concerns

To title:
The title should be changed as no studies were found investigating the long-term effects.**

We have changed the title to “Effect of therapeutic hypothermia on renal and myocardial function in asphyxiated (near) term neonates: a systematic review and meta-analysis**”.**

**To results section:
The text in the results section includes many redundant data which are already provided in Figures 1 and 3. The authors should avoid repeating the same information.**

We shortened the text on the systematic search (lines 167 and 269) as this is already presented in figure 1 and we shortened the legend of figure 3 (line 256).

**Page 9, line 179: the title of table 1 is not complete.**

Thank you for identifying this. We changed “funct” to “function” (line 184).

**Page 12, line 186: the value of the range pH <7.0-7.9 should be checked.**

Thank you for identifying this. That value is indeed false, it should be <7.00-7.09. We corrected this in the manuscript (line 190).

**To discussion section:
As mentioned in general comments, the authors should expand the discussion section about the influence of blood pressure and volume therapy on renal function in asphyxiated neonates treated with TH.**

We added a part to the discussion on factors that may influence renal function and the presence of hypotension in the included studies (line 410)*.*

**Furthermore, they should add some more limitations of the systemic review.
For example, which other factors can influence the renal and myocardial function.**

We added a phrase that other factors may influence the renal and myocardial function, but did not add to much detail because of lack of space (lines 410 and 466).

**Moreover, it may be very helpful for long-term outcome studies, e.g. assessment of myocardial function by echocardiography should be specified.**

In the discussion we point out possible methods to assess renal and cardiac function during the follow-up of asphyxiated neonates (line 497). We added a phrase on that we recommend further research on the best methods to assess renal and cardiac function during this follow-up (line 496).

**In conclusion, the paper presents an interesting review about short-term protective effects of therapeutic hypothermia (TH) on renal and probably myocardial function in asphyxiated full-term neonates. The data can be useful for physicians caring for asphyxiated neonates.**

Thank your very much.

### Reviewer #4 comments:

**Van Wincoop, et al performed a systematic review and meta-analysis of RCT and observational cohort studies of infants with perinatal asphyxia to identify the short and long-term effects of therapeutic hypothermia on renal and myocardial function. They identified 9 studies meeting criteria for short term renal dysfunction (acute kidney injury) and 5 studies meeting criteria for short term myocardial dysfunction. No long-term studies were identified. Meta-analysis found that TH reduced incidence of AKI, with suggestion that TH also was cardioprotective for short term outcomes.

This was a well-written systematic review and meta-analysis of the ongoing problem of multiorgan dysfunction for hypoxic ischemic encephalopathy. It was great that the authors recognize the lack of long-term follow-up studies in this population for systems other than neurodevelopment.**

Thank you for your very helpful comments.

**However, they should further emphasize that therapeutic hypothermia is now considered standard of care in developed countries for moderate to severe HIE, and it will be difficult to compare outcomes to a non-TH group outside of historic controls. We may have a limited window to concurrently investigate longer-term outcomes in children.**

We added a phrase on the fact that TH is now the standard of care and therefore relatively recent studies comparing the effect of TH to a control group are rare (lines 398 and 485).

**One concern with this study is that the rationale for selecting renal and cardiac dysfunction was not well delineated. It seems like the myocardial dysfunction aspect was tacked on and was not as well explored. The manuscript therefore was not as cohesive. Renal and cardiac manifestations after birth asphyxia may be very disparate. In fact, one could argue that cardiac dysfunction could incorporate pulmonary hypertension, poor cardiac output, or evidence of ischemia. Wonder if it would be better to tackle only renal dysfunction in this analysis and focus a separate manuscript on a more detailed cardiac analysis? Or at least justify why these two end-organs were explored and provide more robust assessment of cardiac portion.**

We focused on two organ systems known to be affected by perinatal asphyxia with potential adverse long-term sequelae. We added a phrase to the introduction on why we chose to focus on these two organ systems (line 80). Because there we only a few relevant studies on the effect of TH on cardiac function and the fact that these studies used different cardiac markers and different times of measurement, a more robust analysis on these results was not possible.

**A few other specific comments:

Unclear why search was expanded to include other indications for TH such as near-drowning and aortic arch surgery when inclusion criteria were only for patients in the setting of perinatal asphyxia.**

Although this may seem a bit contradictory, we expanded the third inclusion criterium to also include patients in a setting of near-drowning or aortic arch surgery if not sufficient articles on perinatal asphyxia could be found. We added this to our third inclusion criterium to make this more clear (line 112).

**Long time span of included studies (between 1996-2015). Limitation may be changes in clinical practice over time which could have affected outcomes.**

We agree and added this limitation to our discussion (line 397).

**Good discussion of other limitations- especially heterogeneity of definition of AKI and differences that might be anticipated by including babies with mild HIE into analyses (sensitivity analysis done)**

Thank you.

**As previously mentioned, the myocardial dysfunction discussion needs more detail. Not all studies included all markers for myocardial dysfunction and there seems to be very heterogeneous definition of dysfunction as measured by CK-MB, troponin, BNP, ECG and non-specific ECHO measures in one study, and tissue-Doppler in another study. What is the best marker/definition? Heart failure is different than biochemical or electrocardiographic evidence of ischemia.**

We added a part on the reliability of the used markers to assess cardiac function to the discussion (line 439).

**Results from myocardial dysfunction analysis states “Possible short-term beneficial effects were presented in 4 out of 5 identified studies”. While I understand the descriptive nature given no unified diagnosis of myocardial dysfunction, it is difficult to compare studies. I notice that 4/5 studies did investigate cardiac troponin I levels. Perhaps, at least this measure could have been quantitatively compared between studies?**

Because of the different times of measurements applied in these four studies, no meta-analysis could be performed on the effect of TH on the level of cTnI. We added this explanation of not performing a meta-analysis on the effect of TH on cTnI to the result section (line 298).

**Similarly in the abstract, it states “Possible short-term beneficial effects were presented in 4 out of 5 identified studies” for myocardial function. This is vague and should either be expanded on or left out entirely.**

We expanded this sentence to include how the possible effects were measured (line 49).

**Introduction, line 55. Should read “1 to 2 per 1000” instead of “1 to 2 per 1.000”**

We changed this line to “1 to 2 per 1000” (line 59).
